# Tree Selection of *Vernicia montana* in a Representative Orchard Cluster Within Southern Hunan Province, China: A Comprehensive Evaluation Approach

**DOI:** 10.3390/plants14152351

**Published:** 2025-07-30

**Authors:** Juntao Liu, Zhexiu Yu, Xihui Li, Ling Zhou, Ruihui Wang, Weihua Zhang

**Affiliations:** 1 School of Forestry, Central South University of Forestry and Technology, Changsha 410004, China; ljt1120@bjfu.edu.cn (J.L.); snts123@163.com (X.L.); 2 Key Laboratory of Silviculture and Conservation of the Ministry of Education, College of Forestry, Beijing Forestry University, Beijing 100083, China; yzhexiu@bjfu.edu.cn (Z.Y.); zhouling89757@bjfu.edu.cn (L.Z.); 3 Guangdong Provincial Key Laboratory of Silviculture, Protection and Utilization, Guangdong Academy of Forestry, Guangzhou 510520, China

**Keywords:** *Vernicia montana*, selection of superior trees, comprehensive evaluation method, breeding

## Abstract

With the objective of identifying superior *Vernicia montana* trees grounded in phenotypic and agronomic traits, this study sought to develop and implement a comprehensive evaluation method which would provide a practical foundation for future clonal breeding initiatives. Using the *Vernicia montana* propagated from seedling forests grown in the Suxian District of Chenzhou City in southern Hunan Province, we conducted pre-selection, primary selection, and re-selection of *Vernicia montana* forest stands and took the nine trait indices of single-plant fruiting quantity, single-plant fruit yield, disease and pest resistance, fruit ripening consistency, fruit aggregation, fresh fruit single-fruit weight, fresh fruit seed rate, dry seed kernel rate, and seed kernel oil content rate as the optimal evaluation indexes and carried out cluster analysis and a comprehensive evaluation in order to establish a comprehensive evaluation system for superior *Vernicia montana* trees. The results demonstrated that a three-stage selection process—consisting of pre-selection, primary selection, and re-selection—was conducted using a comprehensive analytical approach. The pre-selection phase relied primarily on sensory evaluation criteria, including fruit count per plant, tree size, tree morphology, and fruit clustering characteristics. Through this rigorous screening process, 60 elite plants were selected. The primary selection was based on phenotypic traits, including single-plant fruit yield, pest and disease resistance, and uniformity of fruit ripening. From this stage, 36 plants were selected. Twenty plants were then selected for re-selection based on key performance indicators, such as fresh fruit weight, fresh fruit seed yield, dry seed kernel yield, and oil content of the seed kernel. Then the re-selected optimal trees were clustered and analyzed into three classes, with 10 plants in class I, 7 plants in class II, and 3 plants in class III. In class I, the top three superior plants exhibited outstanding performance across key traits: their fresh fruit weight per fruit, fresh fruit seed yield, dry seed yield, and seed kernel oil content reached 41.61 g, 42.80%, 62.42%, and 57.72%, respectively. Compared with other groups, these figures showed significant advantages: 1.17, 1.09, 1.12, and 1.02 times the average values of the 20 reselected superior trees; 1.22, 1.19, 1.20, and 1.08 times those of the 36 primary-selected superior trees; and 1.24, 1.25, 1.26, and 1.19 times those of the 60 pre-selected trees. Fruits counts per plant and the number of fruits produced per plant of the best three plants in class I were 885 and 23.38 kg, respectively, which were 1.13 and 1.18 times higher than the average of 20 re-selected superior trees, 1.25 and 1.30 times higher than the average of 36 first-selected superior trees, and 1.51 and 1.58 times higher than the average of 60 pre-selected superior trees. Class I superior trees, especially the top three genotypes, are suitable for use as mother trees for scion collection in grafting. The findings of this study provide a crucial foundation for developing superior clonal varieties of *Vernicia montana* through selective breeding.

## 1. Introduction

Despite being non-renewable, fossil fuels continue to underpin the global economy—a reality that underscores the urgent need to tackle resource depletion through alternative energy solutions [1,2,3,4]. Bioenergy has become an emerging alternative to traditional fossil fuels due to its environmental friendliness and renewability [5,6,7]. As a clean energy source, the application of bioenergy is in line with the concept of sustainable development and circular economy [8,9,10]. With the increasing global demand for energy, bioenergy species have received much attention as an emerging and promising energy resource [11,12,13,14]. However, the yield and fruit contents of bioenergy species are mainly affected by the genetic diversity (genes) of the trees themselves and the environment (pests and diseases, temperature, light) [15,16]. Consequently, the systematic excavation and scientific evaluation of plant resource diversity, in conjunction with the establishment of germplasm repositories tailored to regional environments, are essential prerequisites for the large-scale utilization and industrial upgrading of bioenergy species.

For oily woody plants, the selection of clones with high yield, high quality, and disease resistance is the main goal of breeding programs [17,18,19]. As an important woody oilseed tree species, *Vernicia montana* requires selecting and breeding superior germplasm [20,21]. These superior clonal lines ensure *Vernicia montana* seed yield and quality, improve pest and disease resistance, reduce chemical pesticide use, and safeguard ecological safety [22]. Furthermore, a comprehensive investigation of genetic backgrounds and trait expression mechanisms will not only provide valuable germplasm resources but also establish a theoretical foundation for sustainable development of China’s oil tree industry. Extensive breeding research on *Vernicia montana* in China has demonstrated significant advancements in germplasm characterization and elite cultivar selection. For instance, Zhu et al. [23] systematically collected and characterized Chongqing oleaginous tree germplasm, identifying superior genotypes through integrated analysis of growth traits and economic indicators. Similarly, Li et al. [22] evaluated 14 seed sources by analyzing fruit economic traits, developing a comprehensive scoring model via principal component analysis that identified seven high-performance seed sources. Advanced analytical approaches have been widely applied, as demonstrated by Huang et al. [24], who employed fuzzy mathematics for comparative assessment of germplasm repositories, and Xu et al. [25], who combined typical correlation analysis, ANOVA (one-way analysis of variance), and cluster analysis to select superior *Vernicia montana* genotypes in Hubei while elucidating relationships between growth factors and seed yield. The objective of this study was to develop a phenotypic selection framework to identify elite individual trees of *Vernicia montana* suitable for clonal propagation in southern Hunan Province.

Currently, the selection of superior germplasm resources lacks a comprehensive and systematic evaluation framework despite recent advancements in this field. The comprehensive evaluation method is a multi-criteria decision analysis approach that enables comparative assessment though quantitative scoring based on multiple indicators and weighted factors [15,26]. Due to its advantages of being comprehensive and practical advantages, this method has been shown to significantly improve the efficiency of forest genetic resource utilization [27] and promote sustainable development and precision forestry in modern forestry management. Therefore, the comprehensive evaluation method can be used for superior plant selection and genetic improvement [28,29,30,31].

The millennium tung tree (*Vernicia montana*) exhibits rapid growth, early fruiting phenology, prolonged productive lifespan, high seed oil content [32,33,34], and seed kernel oil content ranging from 39% to 70% [35] and is also a potential bioenergy tree species [36,37]. Tung oil has good water resistance, heat resistance, insulation, and other characteristics and is widely used in industrial fields or used in the manufacture of biodiesel and aviation fuel raw materials. In the selection and breeding of superior varieties of *Vernicia montana*, only Guangxi and Zhejiang provinces in China have selected and bred four asexual lines of the “Gui wrinkle” series and three asexual lines of the “Zhejiang wrinkle” series, respectively. Meanwhile, three high-yielding excellent asexual lines were compared with the reference asexual line in terms of average annual yield: Zhejiang Wrinkle-7, Zhejiang Wrinkle-9, and Zhejiang Wrinkle-8 reached 174.94%, 157.66%, and 137.75% of the reference, respectively [38]. In recent years, the *Vernicia montana* tree has developed rapidly in southern Hunan Province, with total planted area exceeding 20,000 hm^2^. However, the *Vernicia montana* has strong regionality, and all trees are seed-derived, resulting in high genetic variability. As a dioecious species, approximately one-third of the population consists of male individuals, which do not bear fruit. Among female trees, fruiting performance varies due to heterozygosity. These biological factors contribute to low and unstable yields. This tree species displays biological flowering traits—including a short flowering period, a high number of male flowers, and a low female flower ratio—which further contribute to the instability of fruit set. Furthermore, due to the lag in selection and breeding, there is currently no selection and breeding of asexual lines suitable for planting in Hunan Province, China. Therefore, understanding the genetic diversity and relatedness of plants has become crucial for improving fruit yield and maximizing the potential of plants in bioenergy and multi-product development.

This study utilized a 5000 hm^2^ *Vernicia montana* plantation in southern Hunan Province—established through seedling propagation—as the experimental base for elite tree selection. The selection process comprised three phases: (1) primary screening: sixty candidate trees were identified through phenotypic evaluation of morphological traits, including fruit load per plant, tree dimensions, architecture, and fruit clustering patterns; (2) secondary screening: thirty-six superior genotypes were selected based on quantitative production traits (fruit number and yield per plant), agronomic performance (pest/disease resistance, fruit maturation synchrony), and fruit spatial distribution characteristics; and (3) final selection: 20 final elites determined through seed oil production parameters (fresh/dry yield weights and kernel oil content). Using the comprehensive evaluation method, we effectively identified the superior individual *Vernicia montana* plants in southern Hunan Province that possess strong adaptability and high economic value.

## 2. Results

### 2.1. The Selection of Phenotypic Traits for Vernicia montana

Among the five phenotypic traits evaluated, two quantitative factors, fruits per plant and fruit yield per plant, were particularly significant. The 60 selected superior trees exhibited a mean fruit count of 587 fruits per plant and an average yield of 14.87 kg per plant (Table 1). The comprehensive evaluation method [15,26] uses observed trait averages as a reference to determine a scoring system based on mean, standard deviation, and range. Based on this study’s observations, we used 1/10 of the ranges for fruits per plant and fruit yield per plant as grade scores relative to the base value, divided into 10 grades (1–10 points; Table 2). For fruit yield per plant, we established a baseline value of 21.72 kg with a range of 35.66 kg. Using 3.566 kg as the scoring interval, we implemented the following scoring system: yields of 21.72–25.26 kg were assigned six points, 25.29–28.85 kg received seven points, with subsequent intervals following this same incremental pattern.

We used the Kolmogorov–Smirnov test to test the scores of characteristics of the 60 candidate plants (Table 2). The global score followed a normal distribution. Hypothesis testing of sample mean values was performed with the following formula:

(1) $$\begin{array}{c} G=\bar{G}-t_{0.05}\times \frac{\delta }{\sqrt{n}} \end{array}$$

where $\bar{G}$ is the average score of the candidate superior trees (14.71), $t_{0.05}$ is the test value of *p* value at 0.05 level (1.68), *δ* is the standard deviation of the scores of the candidate superior trees (4.39), and n is the number of stands of candidate superior trees (*n* = 60). The calculation result was *G* = 13.76. Based on this scoring, the comprehensive score of candidate superior trees was significantly lower than the average score upper limit of 13.76; thus, among the 60 candidates, those with a comprehensive score > 13.76 were identified as superior trees. Finally, 36 trees were screened, and the selected ratio was 60.00% (Table 3).

The trees with the top three highest scores were GS40, GS8, and GS44 (Table 4). The scores were 29, 27, and 27, respectively. The number of fruits per plant and fruit yield per plant of GS40 was the largest among all the trees. The trees with the lowest score of 14 were GS5, GS18, and GS31. Scores of the 36 selected trees above the mean still showed intra-group differences, indicating that seed-derived offspring of *Vernicia montana* exhibited fruit yield differentiation. Thus, secondary selection was necessary.

### 2.2. Selection of Fruit Quality of Vernicia montana

Fresh fruit mass, fresh fruit seed yield, dry seed kernel yield, and kernel oil yield were used to assess fruit quality, with Table 4 showing the measured mean values, standard deviations, ranges, and base values for the 36 trees.

Following the scoring rules of the comprehensive evaluation method, we established fruit and seed quality scoring standards for *Vernicia montana* (Table 5).

We used the Kolmogorov–Smirnov test to test scores of 36 selected superior trees. The global score followed a normal distribution. Hypothesis testing of sample mean values was based on the formula $G=\bar{G}-t_{0.05}\times \frac{\delta }{\sqrt{n}}$, which resulted in *G* = 19.45. Among the 36 candidate trees evaluated, those with comprehensive scores exceeding 19.45 were advanced to the next selection round. This screening process identified 20 elite genotypes, representing a selection ratio of 55.56% (Table 6).

The trees with the top three highest scores were GS19, GS8, and GS9, with scores of 31, 27, and 27, respectively (Table 6). GS19 demonstrated superior performance, ranking highest in three key yield parameters: fresh fruit seed yield, dry seed kernel yield, and kernel oil yield. GS8 emerged as a top-tier genotype, ranking among the top three in phenotypic trait selection, indicating both optimal morphological characteristics and exceptional fruit quality. Conversely, genotypes GS1, GS12, and GS44 exhibited the poorest performance, with composite scores of 21, 21, and 20, respectively. The scores of the 20 superior trees exceeding the mean still showed intra-group differences, reflecting obvious variations in fruit development and seed quality within *Vernicia montana*. Further screening was therefore necessary.

### 2.3. Cluster Analysis of Screened Superior Vernicia montana Trees

**Figure 1:**
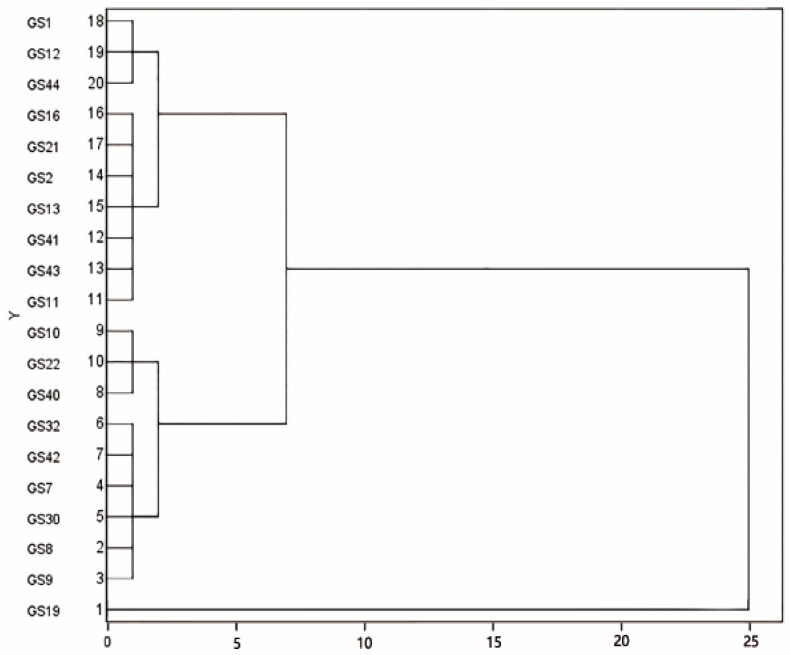
Clustering analysis diagram of 20 superior *Vernicia montana* trees.

Based on the composite scores derived from four key phenotypic traits, we conducted a systematic cluster analysis of the 20 elite genotypes using a comprehensive evaluation approach (Figure 1). Twenty trees were divided into three categories (Figure 1): 10 plants with an average score of 26.20 were in class I (GS19, GS9, GS 8, GS30, GS7, GS42, GS32, GS40, GS22, GS10), which had the best quality fruit. Seven plants with an average score of 22.42 were in class II (GS11, GS43, GS41, GS13, GS 2, GS21, GS16), which had medium fruit quality. Three plants with an average score of 20.66 were in class III (GS44, GS12, GS1), which had relatively poor-quality fruit. Therefore, we focused on class I, especially the top three superior trees in the overall ranking for superior tree selection.

## 3. Discussion

*Vernicia montana* is an indigenous tree species in southern Hunan Province, China [39,40,41]. Long-term natural selection pressures have led to significant phenotypic and genetic differentiation among wild populations. In this study, we evaluated four key fruit quality indicators: fresh fruit mass, seed yield of fresh fruit, kernel yield of dry seed, and oil yield from the kernels. Remarkably, the mean values of these traits in the top three performing genotypes exceeded the population average by 100–200%. Oil yield varied significantly among plants, as most oil tree species exhibit self-incompatibility and high heterozygosity [42,43,44,45]. Current research indicates that seed oil content in *Vernicia montana* follows a polygenic inheritance pattern exhibiting incomplete dominance. Genetic improvement through modulation of fatty acid biosynthesis pathways—particularly via targeted gene editing or transcriptional regulation—can significantly enhance oil quality traits. Furthermore, environmental factors (e.g., climate, soil composition) substantially influence phenotypic expression of these oleaginous characteristics in forest trees. The three superior *Vernicia montana* trees all came from the same slope with a small variation. The slope was gentle with fertile soil, good drainage, and sufficient light. Therefore, for the selected *Vernicia montana* superior trees with good fruit and seed quality, we should evaluate the performance further by a regional planting test that could cover a large planting range and different site conditions. Oil production of oil trees is related to cultivation technology. The growth condition of the tree can affect the phenotypic and economic quality of the fruit. Using high-density planting of dwarf *Vernicia montana* can improve labor efficiency and reduce production cost. The phenomenon of the biennial bearing of *Vernicia montana* is obvious. The results from an “on” year can more easily reflect the potential fruit yield of trees [46]. Our selection was carried out in an “on” year [22], which effectively avoided the impact of the “off” year on selection.

Our results demonstrate significant variation in both fruit yield and quality among *Vernicia montana* individuals, consistent with the patterns observed in superior tung tree stands by Zhou and Deng [27]. After the evaluation of tung trees, Liu [47] obtained three superior lineages with high, stable yield, high-quality fruit, and adaptability, which greatly improved the fruit yields of tung trees from 40% to 70% in Henan. Wang and Song [48] conducted an experiment to investigate the yield structure, fruit quality, branch traits, sequences of flowers and fruits, tree structure, and oiliness physicochemical properties of selected trees from Guo’s study, and they screened five plants that had an average yield that was 67 % higher than the control for this high-yielding lineage [38]. *Vernicia montana* has a strong regional character. In the 1980s, Wang et al. [48] systematically performed the selection of superior *Vernicia montana* trees and identified “GZ27”, “ZZ7”, and another clones. The three selected plants had increased yields of 50–65% compared with existing varieties [38]. These findings suggest that genotype selection should be prioritized when establishing *Vernicia montana* plantations, with careful matching of elite individuals to local growing conditions. In recent years, China has conducted extensive research on breeding conventional oilseed tree species, achieving significant progress in selecting and breeding superior individual plants. Candidate selection prioritized vegetative traits (growth form, disease resistance) and reproductive traits (seed oil content, fruit yield) per standard economic tree improvement protocols [49,50,51]. For example, for woody oilseed species such as *Xanthoceras sorbifolium*, *Sapindus indica*, and *Vernicia montan* [27], the selection of good monocots of these species will provide a certain scientific basis for the cultivation and propagation of good asexual lines of each species.

As far as selection methods are concerned, the methods for selecting superior trees include the independent criterion method, continuous selection method, and comprehensive scoring method [52]. The independent standard selection method for elite genotype identification requires candidates to surpass established threshold values across all target traits simultaneously. While this approach simplifies and clarifies decision-making, its main limitation is stringent all-or-nothing criteria: individuals excelling in multiple traits are rejected if they fail any single evaluation threshold. The successive selection method necessitates multi-year evaluations, resulting in extended time requirements and substantial resource investments. Consequently, this approach is only practical for a limited number of tree species in elite genotype selection programs. The comprehensive scoring method is a subjective weighting approach. While influenced by human factors, it can reasonably determine the importance of each indicator, thereby screening out *Vernicia montana* specimens that meet the energy tree species goals [53]. The scoring method is to take the average value of each trait of the candidate superior tree as a reference for grading scoring and to determine the scoring system for selection of superiority based on the mean, standard deviation, and extreme deviation of each trait, which can comprehensively take into consideration of multiple traits, effectively avoiding the problem of large bias in the evaluation of selection indexes [52]. In recent years, the comprehensive scoring method has been widely used in the selection of superior trees of most tree species, such as *Cornus walteri* Wanger [54], *Cinnamomum longepaniculatum* [55], *Moringa oleifera* [52], *Lyciumruthenicum* [56], and other tree species, since most of the tree species in the selection process involve a large number of indicators of excellence, the differences are significant, and the use of the comprehensive scoring method of the selection of superior trees of a large number of tree species has reached a good effect for excellence selection. The results of this experiment show that selected superior *Vernicia montana* trees in class I from this study have great potential for popularization and utilization. Further research on the breeding of *Vernicia montana* should be conducted. Scions from the superior trees in class I and rootstocks from *Vernicia montana* seedlings can be used for grafting. After 1 year of cultivation, they can be used for forestation [57]. To enhance propagation efficiency, we recommend establishing a dedicated cutting orchard using grafted seedlings, which ensures sustainable scion supply. For existing male *Vernicia montana* specimens in production forests, yield improvement can be achieved through top-grafting with either class I superior tree scions or verified high-performance clones from the cutting orchard.

As a typical insect-pollinated tree species, the reproductive biology of the *Vernicia montana* exhibits significant outcrossing adaptation advantages [58,59]. Compared to free pollination, hybrid breeding has a significant synergistic effect [60,61]. By artificially controlling pollen sources, the range of gene exchange can be expanded, thereby enhancing pollination heterozygosity. Studies have shown that bee pollination treatments can significantly increase fruit set rates compared to natural pollination, thereby increasing fruit thousand–grain weight. Additionally, hybrid pollination optimizes the pollen competition environment, significantly enhancing the growth rate of heteropollen tubes and significantly increasing the fertilization opportunities for high-quality pollen. The top three superior genotypes identified in this study exhibited high single-plant yields and large fruit sizes. These high-yield traits may be closely associated with pollen vitality, as previously observed in Vernicia and Diospyros species [22,60]. This highlights the effectiveness of outcrossing in enhancing productivity in woody oilseed crops.

This study utilized a phenotypic evaluation approach to systematically assess individual *Vernicia montana* trees through a multi-indicator scoring system. The analysis preliminarily identified elite genotypes exhibiting superior traits, including high single-tree yield, enhanced disease resistance, and elevated seed oil content. However, this method also has certain limitations, such as failing to consider genotype information and long-term environmental stability, and the results may be influenced by factors such as annual climate fluctuations. Therefore, in subsequent studies, it is necessary to combine molecular marker technology and long-term multi-site validation methods to further improve breeding precision. Nevertheless, the superior genotypes selected in this study demonstrate consistent performance across multiple agronomic traits, indicating strong potential for practical applications. On one hand, high-yielding clonal lines can be established through asexual propagation methods such as grafting, providing high-quality seed sources for energy forest planting in regions like Hunan. On the other hand, these materials can also serve as core foundational materials for subsequent genetic diversity studies and functional gene discovery, providing support for the efficient utilization and industrial development of the *Vernicia montana*.

As an important economic tree species, the fruit yield of the *Vernicia montana* directly determines the oil production per unit area, while the oil content of the seeds influences the quality of the oil and economic benefits. Therefore, fruit yield and oil content are the core indicators for assessing the economic value of the *Vernicia montana*. This study focuses on phenotypic characteristics directly related to economic traits to guide the selection of superior individuals. However, reproductive biological characteristics such as sex ratio, flowering synchrony, flower morphology, and pollination mechanisms significantly influence fruit formation and yield. Although we fully recognize the importance of these traits, due to research resource and time constraints, as well as the need for long-term, systematic field observations and experiments (such as flower morphology measurements and pollinator tracking), this study was unable to concurrently conduct related work. Additionally, the relationship between reproductive traits and fruit yield is complex, often influenced by multiple factors such as environmental conditions and pollinator abundance, making it difficult to establish clear quantitative relationships in the short term, thereby limiting their application in the selection of superior trees at this stage. Nevertheless, we will prioritize the reproductive biology of the *Vernicia montana* in future studies, systematically investigating aspects such as sex ratio, flowering synchrony, floral structure, and pollination mechanisms. This will provide theoretical support for elucidating the mechanisms underlying fruit trait formation and optimizing breeding standards.

## 4. Materials and Methods

### 4.1. Overview of the Experimental Site

**Figure 2:**
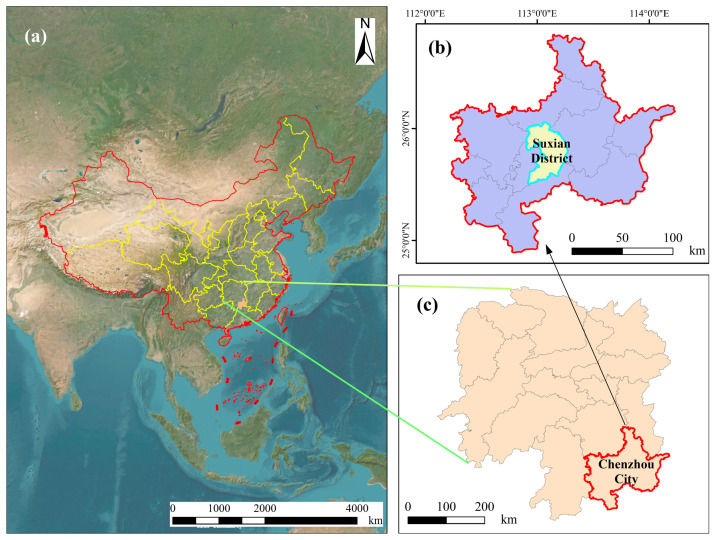
Map of the study area. (**a**) China map. (**b**) Chenzhou City map. (**c**) Hunan Province map.

**Figure 3:**
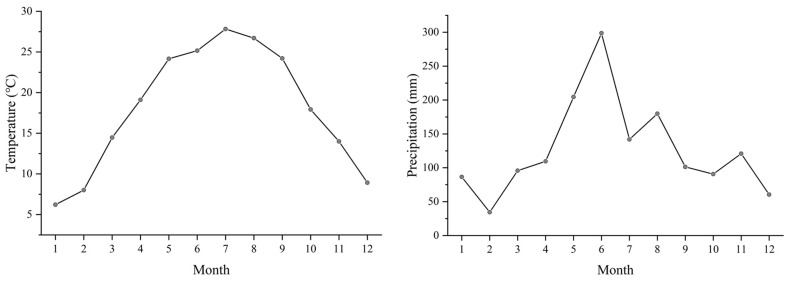
The average monthly temperatures and average precipitation of Suxian District in 2018. Note: The broken lines with circles indicate temperature and precipitation respectively.

The experimental site is located in Suxian District, Chenzhou City, Hunan Province, China (24°53′–25°41′ N, 112°37′–113°20′ E) (Figure 2). The elevation ranges from 104 to 1913.8 m. The region has a subtropical humid monsoon climate, with an annual average temperature of 18.0 °C, annual precipitation of approximately 1444.5 mm (Figure 3), and relative humidity consistently around 78%. The cumulative temperature throughout the year is relatively high, with precipitation concentrated in the summer. The annual maximum temperature is 34 °C, and the minimum temperature is 3 °C. The seasons are distinct, with a longer summer, shorter autumn, and mild winter. The soil types in the experimental area are primarily red soil or yellow soil. The parent material of the soil is mainly sandstone and limestone, with a heavy texture and a neutral to slightly acidic pH of approximately 5.42. The soil fertility is good, with total carbon at 7.35 g/kg, total nitrogen at 0.8 g/kg, total phosphorus is 0.14 g/kg, and total potassium is 6.06 g/kg. The forest land is relatively flat, with an elevation of 325 m, and there is no significant shading effect, making it suitable for the growth of *Vernicia montana*. Most *Vernicia montana* trees in the experimental area were planted along contour lines, with row spacing of 3–4 m, slope of 15°–30°, canopy density of 0.5–0.8, and understory coverage of 40%–80%. All experimental trees were planted in the same year (2010) and managed consistently. No significant competitive tree species or vine interference was observed. The main shrubs and herbaceous plants include sedge, burdock, black nightshade, camellia, and scale fern.

### 4.2. Materials

**Figure 4:**
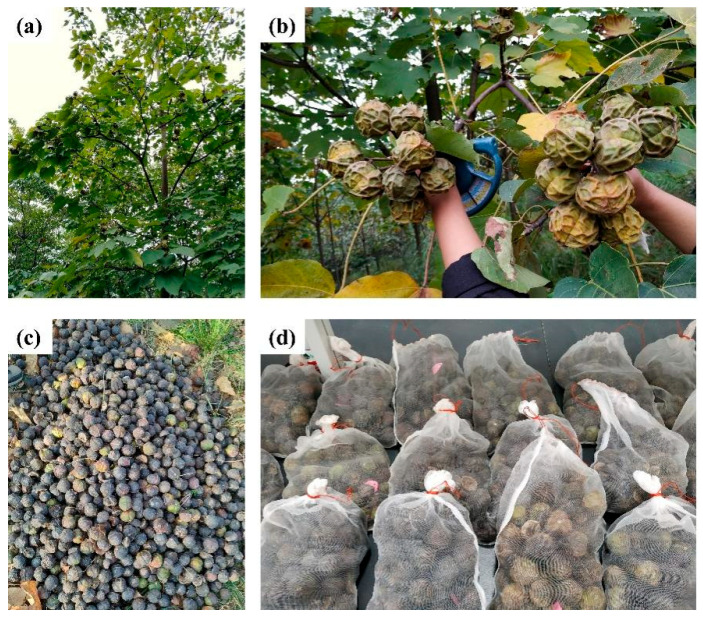
Field experiment work photos. (**a**) The *Vernicia montana* of experimental field. (**b**) The process of fruit collection. (**c**) The fruit was collected from a single tree. (**d**) The fruit was harvested and subsequently subjected to experimentation within an indoor setting.

**Figure 5:**
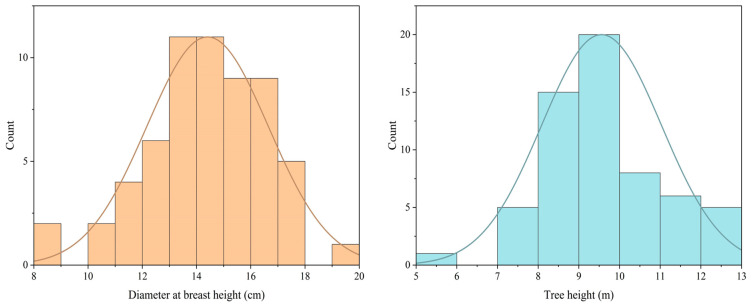
The frequency distribution diagram of breast height diameter and tree height.

The experimental material consists of *Vernicia montana* established through seedling afforestation using seeds collected from the same batch in 2010 by Chenzhou Guosheng Bio-Energy Co., Ltd. from Suining County, Hunan Province (China). The selection of superior *Vernicia montana* was primarily conducted in Suxian District, Chenzhou City (Figure 4). The trees are 8 years old, with an average height of 9.56 m and an average breast height diameter of 14.41 cm (Figure 5). The forest stand exhibits optimal management practices and vigorous growth characteristics, having reached peak fruit production, which qualifies it as valuable germplasm for novel cultivar breeding programs.

### 4.3. The Selection of Superior Trees

The selection and investigation of superior trees followed the following principles: (1) During 2016–2017, the selection criteria for optimal *Vernicia montana* (millennium tung) trees were established based on the uniform tree age, homogeneous site conditions, consistent forest structure, and vigorous growth performance. (2) After selecting the optimal forest stand, it was surveyed on foot to assess its condition and vegetation. After 2–3 years of observation, 32 trees were initially selected. In the fall of 2018, we visually inspected the trees every two rows along the planting line from the top of the slope to the bottom of the slope and selected 28 trees. A total of 60 candidate superior trees were selected. Following identification, candidate trees were permanently marked at breast height (1.5 m) using numbered spray paint for long-term monitoring and tracking. (3) The superior tree selection protocol included comprehensive biometric measurements (height, DBH, crown width) and ecological assessments of stand conditions (stand age, density, canopy cover, understory vegetation) and site characteristics (slope gradient, aspect, elevation, soil properties). During fruit maturation, we conducted individual tree harvests to quantify both fruit number and yield per plant. A total of 60 candidate superior trees were selected, characterized by straight trunks, heavy fruiting, high fruit aggregation, distinct branching, large fruits, and no obvious pests or diseases. After the candidate superior trees were determined, a number was painted on the selected tree at 1.5 m height. Tree height and DBH were measured. After the fruit matured, we harvested them from each ramet separately. Using five traits, (i.e., the number of fruits per plant, fruit yield per plant, resistance to pests and diseases, consistency of fruit ripeness, and the degree of fruit agglomeration), we selected 36 superior trees from the original 60. Based on comprehensive evaluation of four key metrics—fresh fruit mass, seed yield per fresh fruit, kernel yield from dry seeds, and kernel oil extraction yield—we identified and selected the top 20 superior genotypes. Pest and disease resistance (scoring criteria: no obvious pests and diseases = 4 points, mild pests and diseases = 3 points, moderate pests and diseases = 2 points, serious pests and diseases = 1 point), fruit ripeness consistency (scoring criteria: ripeness ratio 80% or more = 4 points, ripeness ratio 60–80% = 3 points, ripeness ratio 60–40% = 2 points, ripeness ratio 40% or less = 1 point), and aggregated fruit count (scoring criteria: number of fruits 7 or more = 4 points, number of fruits 5–7 = 3 points, number of fruits 3–4 = 2 points, number of fruits 1–2 = 1 point) were qualitative indexes, and pest and disease resistance, fruit ripeness consistency, and aggregated fruit count were scored on a scale of 1 to 4, scored based on observed phenotypic traits (Table 7).

### 4.4. Sample Collection and Determination of Traits

We collected fruits from 60 selected *V. montana* accessions at physiological maturity (25 October 2018), with subsequent harvest of fully ripened fruits from potential superior genotypes. First, the total number of fruits per plant was recorded, and then the fruits were thoroughly mixed. Subsequently, 1.5 kg of fruits were randomly sampled from each test plant, sealed in airtight bags, labeled with records and numbers, and transported back to the laboratory. From the 1.5 kg of sample fruits, 15 were randomly selected, weighed and recorded as the total mass of fresh fruits. The shells of the 15 fruits were then peeled off and the seeds were removed and weighed and recorded as the mass of fresh seeds. The fresh seeds were dried to a constant mass and recorded as dry seed mass. The dried seeds were peeled and the kernels were removed and weighed and recorded as the mass of the dried kernels. The traits we used to select trees were determined based on previous studies [23,27,62,63]. The determination method refers to the GB/T144488.1-2008 determination of oil content in oil plants [63]. Take the dried seeds of the thousand-year-old tung tree, dry them, and weigh them with an accuracy of 0.001 g. Grind the dried seeds into powder, then use an electronic balance to weigh out 4.5 g of the seed kernels. Wrap the weighed sample in qualitative filter paper (M _filter paper_) to form a sample packet. Place the sample packet in the center of a petri dish and place it in a drying oven set to 105 °C. Dry in the oven for 2 h, remove the sample, and weigh the sample mass (m_1_) using an electronic balance. Place the sample in an extraction flask, add 70–80 mL of petroleum ether, and extract using a Soxhlet extractor at 60 °C for 6–12 h. Turn on the condenser water switch to establish a steady flow of condenser water, then proceed with the extraction in a constant-temperature water bath. The extraction is deemed complete once the sample bag has been placed in the extraction flask and no residual oil remains in the bag. Following valve closure, remove the sample bag with sterile tweezers and oven-dry at 105 °C until constant weight (m_2_). Seed kernel oil content was derived gravimetrically. All traits were measured in triplicate using an analytical balance with a precision of ±0.01 g. The following formulas were applied for quantitative analysis:

(2) $$\begin{array}{c} M_{FF}=\frac{M_{TF}}{N_{FF}}\times 100\% \end{array}$$

where $M_{FF}$ is fresh fruit mass, $M_{TF}$ is total fresh mass of fruits, $N_{FF}$ is number of fruits;

(3) $$\begin{array}{c} SY_{FF}=\frac{M_{TS}}{M_{TF}}\times 100\% \end{array}$$

where $SY_{FF}$ is seed yield of fresh fruit, $M_{TS}$ is total fresh mass of seeds, $M_{TF}$ is total fresh mass of fruits;

(4) $$\begin{array}{c} {KY}_{DS}=\frac{M_{KDS}}{M_{DS}}\times 100\% \end{array}$$

where ${KY}_{DS}$ is kernel yield of dry seed, $M_{KDS}$ is kernel mass of dry seeds, $M_{DS}$ is dry seed mass;

(5) $$\begin{array}{c} {OY}_{K}=\frac{M_{ODK}}{M_{DK}}\times 100\% \end{array}$$

where ${OY}_{K}$ is oil yield from the kernels, $M_{ODK}$ is mass of oil from dry kernels, $M_{DK}$ is dry kernel mass.

### 4.5. Statistics and Analysis of Data

Excel 2003 (USA) and SPSS 21.0 (USA) were used for data analysis. Graphs were drawn using Origin 2021pro (USA). Composite scores were performed and ranked after using the K-S test. The scoring standards for each trait were established based on three statistical parameters: the mean, standard deviation, and range of observed values. For non-quantifiable and non-gradable traits, a comprehensive scoring system for selecting superior *Vernicia montana* was developed to screen elite individual plants with high yield, high quality, and pest/disease resistance [27,54], that is, on the basis of the base value, with 10 points divided into grades and assigned 1 to 10 points to establish the scoring standard of each trait marker. The t-test was used to determine the significant lower limit value below the mean value of the population of candidate superior trees, that is, the minimum score value for superior tree selection. We used SPSS software to perform cluster analysis on 20 superior *Vernicia montana* trees, selecting appropriate distance measurement methods (such as Euclidean distance) and using intergroup linkage analysis in Euclidean distance.

## 5. Conclusions

This study employed a comprehensive evaluation method and cluster analysis to evaluate nine observational indicators of 60 candidate superior trees. Based on phenotypic indicators such as single-tree fruit yield, single-tree fruit production, disease and pest resistance, fruit ripening consistency, and fruit clustering, 36 preliminary superior trees were selected. With regard to indicators such as fresh fruit single fruit weight, fresh fruit seed yield rate, dry seed kernel yield rate, and seed kernel oil content rate, 20 superior trees were selected. We further conducted cluster analysis on the 20 selected superior trees, dividing them into three categories: 10 trees in class I, 7 in class II, and 3 in class III. The average fresh fruit weight, fresh fruit seed yield, dry seed kernel yield, and kernel oil yield of class I trees were 13.83 g, 41.03%, 59.85%, and 57.03%, respectively, which were 1.03, 1.04, 1.07, and 1.01 times the average values of the 36 trees selected in the second step and 1.08, 1.14, 1.15, and 1.07 times the average values of the 60 trees in the first step, respectively. The top three superior trees in class I were GS19, GS8, GS9. Among the top three trees in class I, the three outstanding trees have characteristics such as high single-tree fruit yield, numerous clusters, and high seed kernel oil content and can serve as mother trees for future selection and breeding of superior *Vernicia montana* varieties. This paper preliminarily established an evaluation index system for selecting superior *Vernicia montana* using a comprehensive evaluation method, thereby providing a scientific basis for establishing Vernicia montana nurseries and efficiently cultivating clonal varieties. Additionally, the selection criteria for superior *Vernicia montana* proposed in this study are only applicable to the distribution area of *Vernicia montana* in Chenzhou, Hunan Province, and may not be representative of other provinces. Their applicability requires further verification in practice.

## Figures and Tables

**Table 1:** The mean value, standard deviation, range, and base value of the measured phenotypic traits of 60 *Vernicia montana* trees.

Phenotypic Trait	Mean	Standard Deviation	Range	Base Value
The no. of fruit per plant (count)	587	282	1346	869
Fruit yield per plant (kg)	14.87	6.85	35.66	21.72

**Table 2:** Scoring standards of the phenotypic traits of *Vernicia montana* trees based on the comprehensive evaluation method.

The No. of Fruits per Plant		Fruit Yield per Plant	
Base Value (Count)	Score	Base Value (kg)	Score
196~330.6	1	3.89~7.46	1
330.6~465.2	2	7.46~11.02	2
465.2~599.8	3	11.02~14.59	3
599.8~734.4	4	14.59~18.15	4
734.4~869	5	18.15~21.72	5
869~1003.6	6	21.72~25.29	6
1003.6~1138.2	7	25.29~28.85	7
1138.2~1272.8	8	28.85~32.42	8
1272.8~1407.4	9	32.42~35.98	9
1407.4~1542	10	35.98~39.55	10

**Table 3:** Evaluation of *Vernicia montana* phenotypic traits.

No. of the Candidate Tree	The No. of Fruits per Plant (Count)	Fruit Yield per Plant (kg)	Resistance to Pests and Diseases	Consistency of Fruit Ripeness	The No. of Fruits in Aggregation (Count)	Total Score
GS1	3	3	3	4	2	15
GS2	2	2	3	4	3	14
GS5	2	3	4	3	2	14
GS6	4	4	3	4	2	17
GS7	3	4	3	3	2	15
GS8	9	9	3	3	3	27
GS9	4	5	2	4	2	17
GS10	3	5	2	3	3	16
GS11	5	4	3	3	2	17
GS12	8	7	3	4	2	24
GS13	5	5	2	2	1	15
GS16	3	4	3	4	1	15
GS18	1	2	3	4	4	14
GS19	4	4	4	4	2	18
GS21	7	4	3	2	2	18
GS22	5	3	2	3	4	17
GS32	2	2	4	4	3	15
GS25	3	3	4	4	3	17
GS27	5	6	2	4	1	18
GS28	3	5	2	3	3	16
GS29	3	4	2	4	3	16
GS30	2	4	4	4	1	15
GS31	3	3	2	4	2	14
GS32	4	5	2	4	2	17
GS37	3	4	4	2	2	15
G382	4	4	3	4	1	16
GS40	10	10	3	4	2	29
GS41	4	6	2	3	1	16
GS42	4	5	4	3	2	18
GS43	5	5	1	2	4	17
GS44	8	8	4	4	3	27
GS45	6	6	1	1	4	18
GS46	4	4	2	3	3	16
GS47	4	4	2	4	2	16
GS56	6	4	3	2	2	17
GS59	3	3	4	2	3	15

Note:GS1–GS60 are superior *Vernicia montana* trees.

**Table 4:** Mean, standard deviation, range, and the base value of the fruit and seed quality of 36 selected superior trees of *Vernicia montana*.

Phenotypic Trait	Mean	Standard Deviation	Range	Base Value
Fresh fruit mass (g)	34.22	8.88	30.39	43.10
Seed yield of fresh fruit (%)	35.92	7.78	34.17	43.70
Kernel yield of dry seed (%)	51.82	8.93	42.38	60.75
Oil yield from the kernels (%)	53.50	7.15	36.22	60.65

**Table 5:** Scoring standards for fruit and seed quality traits of superior trees of *Vernicia montana* based on the comprehensive evaluation method.

Fresh Fruit Mass		Seed Yield of Fresh Fruit		Kernel Yield of Dry Seed		Oil Yield from the Kernels	
Base Value (g)	Score	Base Value (%)	Score	Base Value (g)	Score	Base Value (%)	Score
18.78~21.82	1	23.19~26.65	1	31.08~35.32	1	35.29~38.92	1
21.82~24.86	2	26.65~30.03	2	35.32~39.56	2	38.92~42.54	2
24.86~27.90	3	30.03~33.45	3	39.56~43.80	3	42.54~46.16	3
27.90~30.94	4	33.45~36.87	4	43.80~48.04	4	46.16~49.78	4
30.94~33.98	5	36.87~40.28	5	48.04~52.27	5	49.78~53.41	5
33.98~37.02	6	40.28~43.70	6	52.27~56.51	6	53.41~57.03	6
37.02~40.06	7	43.70~47.12	7	56.51~60.75	7	57.03~60.65	7
40.06~43.1	8	47.12~50.54	8	60.75~64.99	8	60.65~64.27	8
43.1~46.13	9	50.54~53.95	9	64.99~69.23	9	64.27~67.89	9
46.13~49.19	10	53.95~57.37	10	69.23~73.46	10	67.89~71.52	10

**Table 6:** Summary of the comprehensive evaluation of the fruit and seed quality of 20 superior *Vernicia montana* trees.

No. of the Selected Tree	Score of Fresh Fruit Mass	Score of Seed Yield of Fresh Fruit	Score of Kernel Yield of Dry Seed	Score of Oil Yield from the Kernels	Total Score
GS1	3	6	4	8	21
GS2	1	7	7	7	22
GS7	7	5	7	7	26
GS8	9	3	8	7	27
GS9	10	5	7	5	27
GS10	8	4	6	6	24
GS11	7	4	6	6	23
GS12	3	6	7	5	21
GS13	6	6	6	4	22
GS16	9	6	2	5	22
GS19	4	10	9	8	31
GS21	9	5	3	5	22
GS22	2	9	8	5	24
GS30	3	7	7	9	26
GS32	3	7	10	6	26
GS40	8	2	7	8	25
GS41	9	1	6	7	23
GS42	10	5	5	6	26
GS43	8	4	5	6	23
GS44	3	2	5	10	20

**Table 7:** Scoring standards of resistance to pests and diseases, consistency of fruit ripeness, and the number of fruits in aggregation for *Vernicia montana* trees.

Selected Phenotypic Trait	Characteristic	Score	Characteristic	Score	Characteristic	Score	Characteristic	Score
Resistance to pests and diseases	no obvious pests and diseases	4	mild pests and diseases	3	moderate pests and diseases	2	serious pests and diseases	1
Consistency of fruit ripeness	80% or more	4	60–80%	3	60–40%	2	40% or less	1
The no. of fruits in aggregation	7 or more	4	5–7	3	3–4	2	1–2	1

## Data Availability

No new data were created or analyzed in this study. Data sharing is not applicable to this article.
